# Supplementary material for: Combined Bulked Segregant Analysis-Sequencing and Transcriptome Analysis to Identify Candidate Genes Associated with Cold Stress in Brassica napus L
Source: Int J Mol Sci. 2025 Jan 28;26(3):1148. doi: 10.3390/ijms26031148 (PMC11818577; doi:10.3390/ijms26031148)
Supplement: Supplementary file 1 [file ijms-26-01148-s001.zip › Supplementary Figure_jjy.pdf]

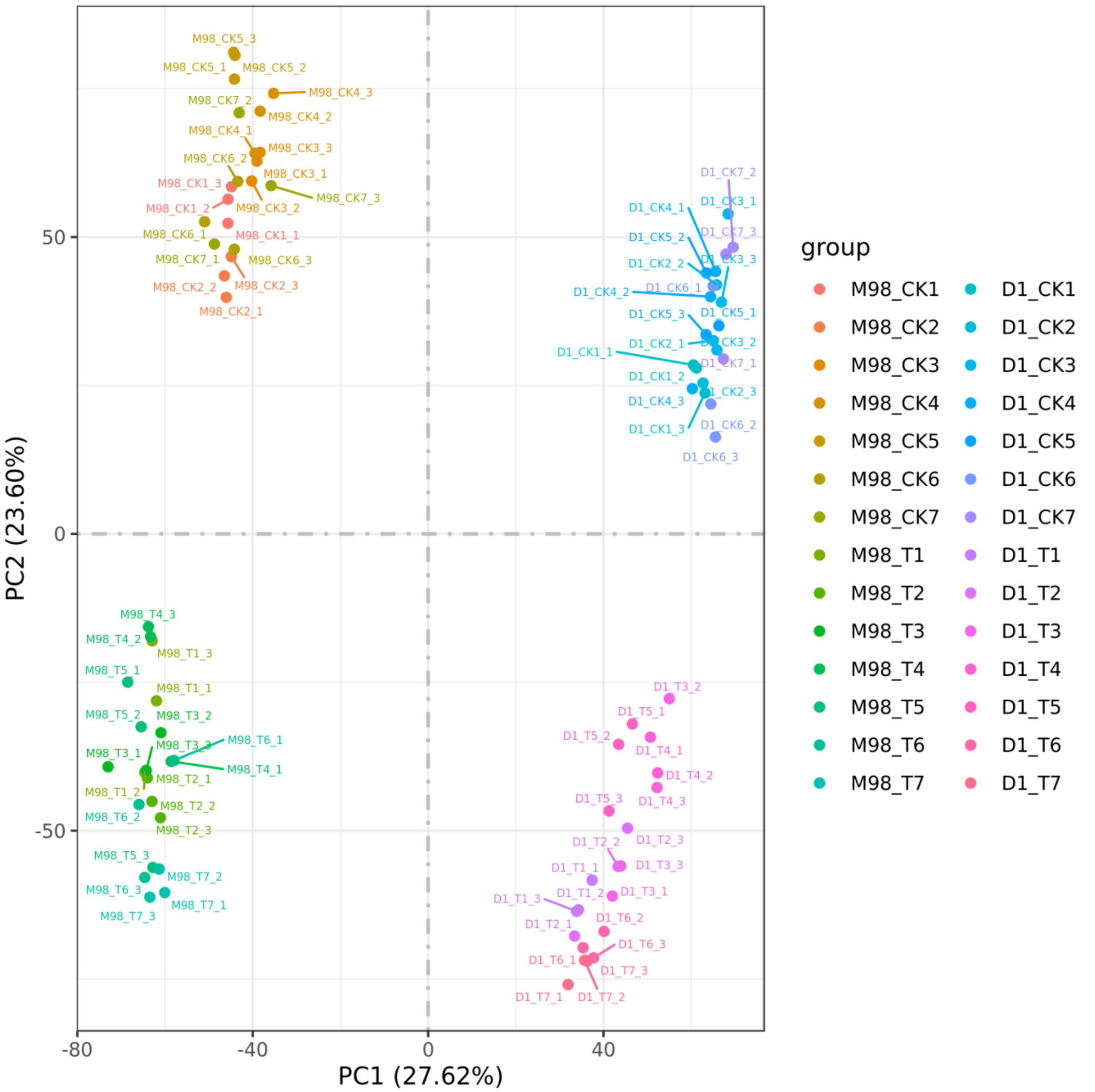

Supplementary Figure S1 Principal component analysis (PC) of all samples. Different colors represent samples at different temperature treatment time points. CK: control; T, low-temperature treatment.

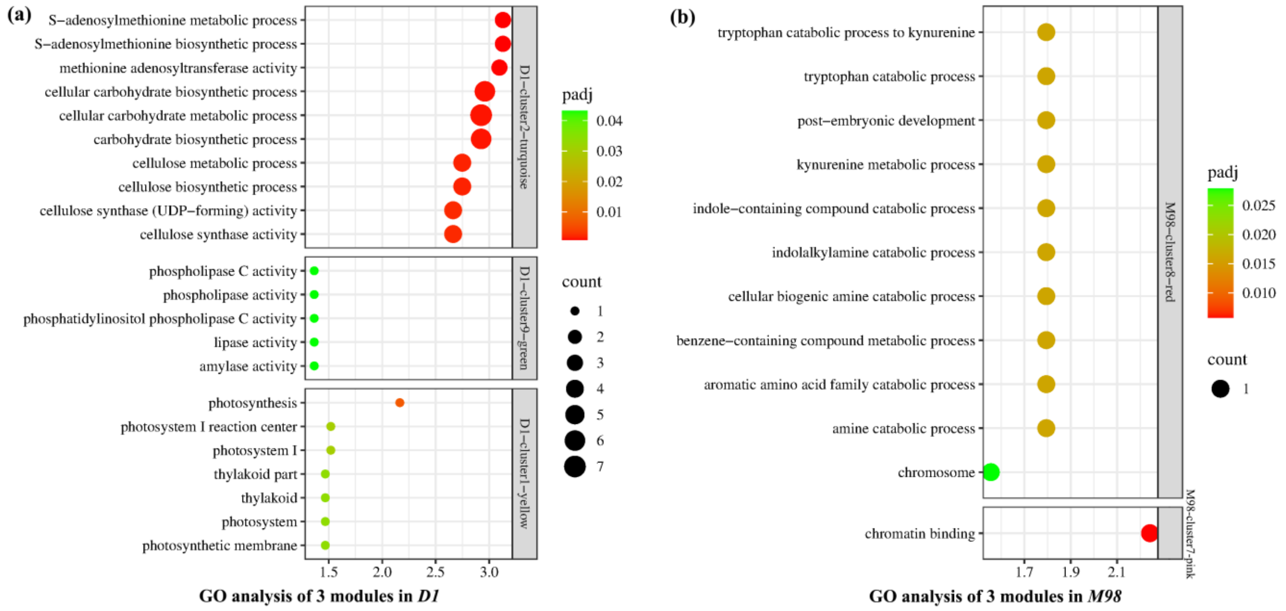

Supplementary Figure S2 GO enrichment analysis of six new modules. GO analysis of 3 modules in *D1* (a) and in *M98* (b). The X-axis represents -log10(Padj) and enriched GO terms are indicated on the Y-axis.

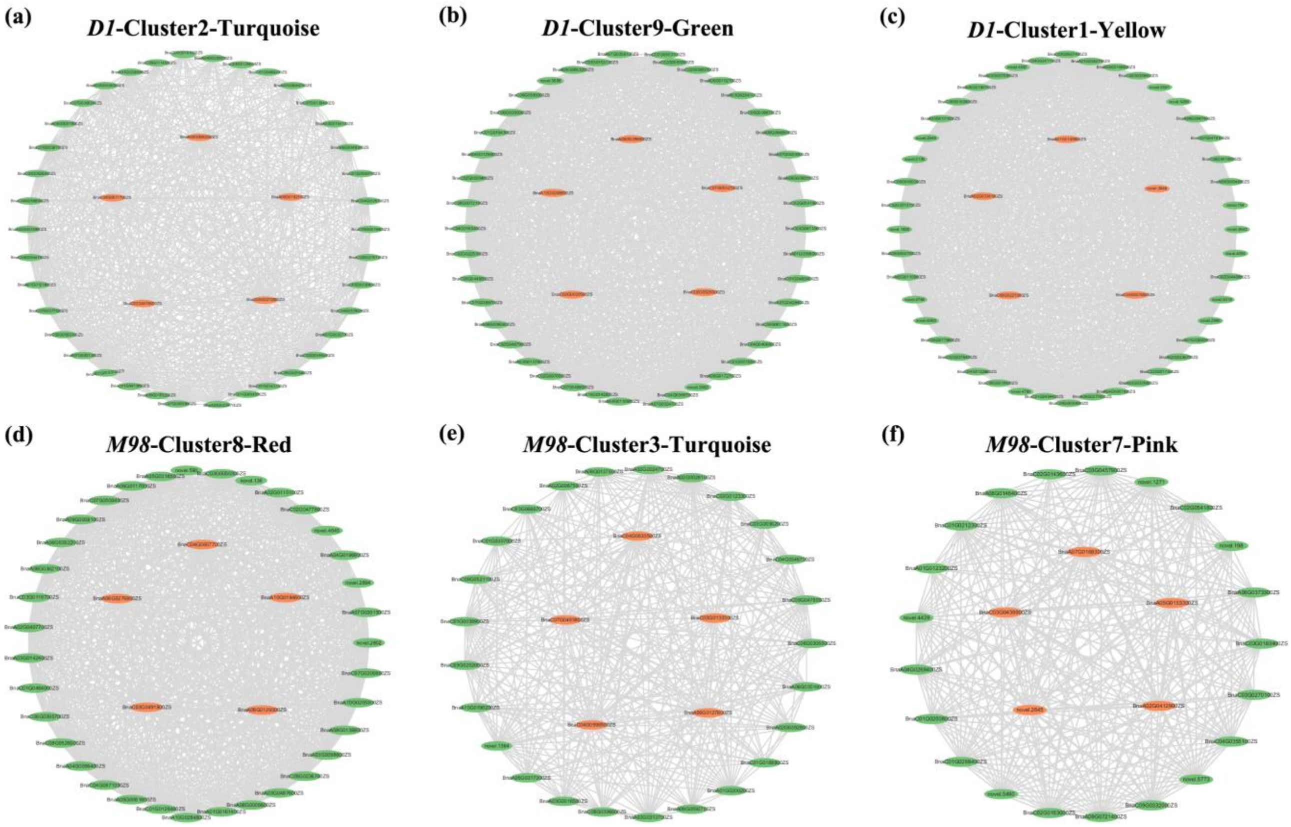

Supplementary Figure S3 Gene co-expression correlation networks for six new modules. Gene co-expression correlation networks for D1-Cluster2-Turquoise (a), D1-Cluster9-Green (b), D1-Cluster1-Yellow (c), M98-Cluster8-Red (d), M98-Cluster3-Turquoise (e), N98-Cluster7-Pink (f). Hub genes are indicated by orage circles; Green circles indicate associated genes.

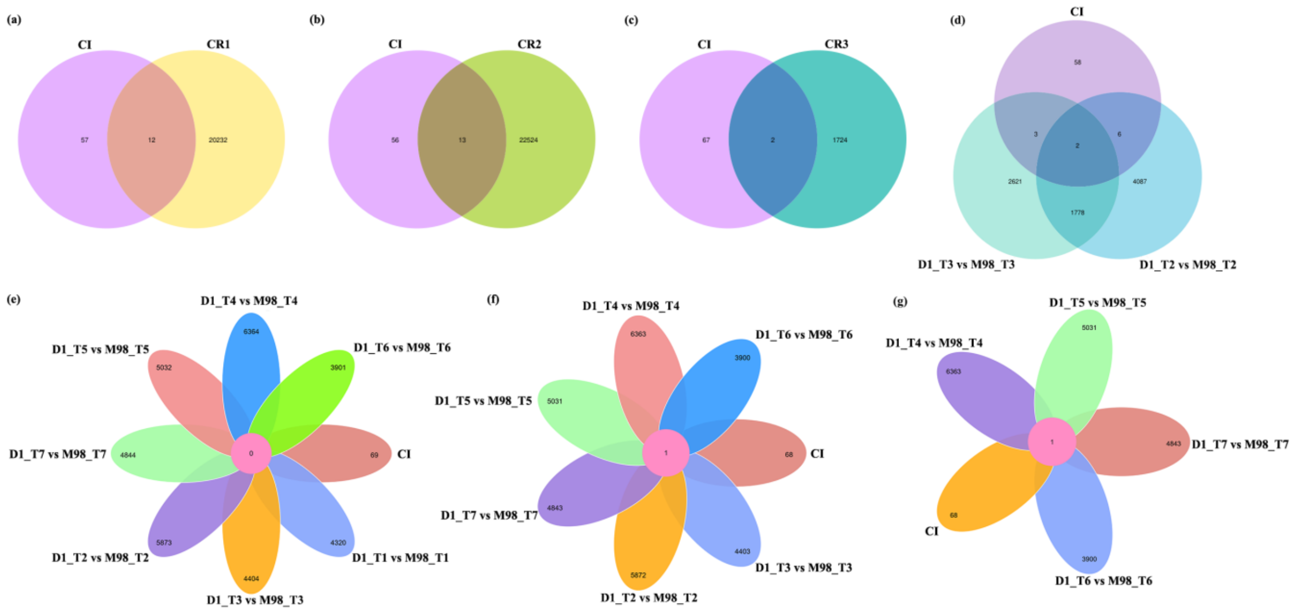

Supplementary Figure S4 Veen analysis. The intersections genes of CI and CR1 (a), CI and CR2 (b), CI and CR3 (c), D1_T1 vs. M98_T1 to D1_T7 vs. M98_T7 and CI (e), D1_T2 vs. M98_T2 to D1_T7 vs. M98_T7 and CI (f), D1_T2 vs. M98_T2 to D1_T3 vs. M98_T3 and CI (d), D1_T4 vs. M98_T4 to D1_T7 vs. M98_T7 and CI (g), respectively. CI, the candidate interval of BSA-seq. D1_T1 vs. M98_T1, a gene set after removing D1_CK1 from the D1_T1 vs. a gene set after removing M98_CK1 from the M98_T1. The remaining differential groups were similarly handled, with the corresponding expression genes in CK also excluded. CK: control; T, low-temperature treatment.

```
BnaC09G0354200ZS_M98    ATGATGGAAGAATATATTGATTTTCGACCACTGAAATACACAGAGCACAAAACATCAGTCACTAAATACACCAAAAAGTCACCGGCAAAAAATCCCTCCG   100
BnaC09G0354200ZS_D1     ATGATGGAAGAATATATTGATTTTCGACCACTAAAATACACAGAGCACAAAACATCAGTCACTAAATACACCAAAAAGTCACCGGCAAAAAATCCCTCCG   100

BnaC09G0354200ZS_M98    GCGAAACTCGACCTGACTCAGTGAGAATCGTTCGTGTCTCTGTAATCGATCCTTACGCAACCGATTCATCAAGTGACGAAGAAGACTTCCTCTTTCCTCG   200
BnaC09G0354200ZS_D1     GCGAAACTCGACCTGACTCAGTGAGAATCGTTCGTGTCTCTGTAACCGATCCTTACGCAACCGATTCATCAAGTGACGAAGAAGACTTCCTCTTTCCTCG   200

BnaC09G0354200ZS_M98    CCGGCGAGTCAAGAGATTCGTTAACGAGATCAAAGTCGAGCCAGCCTGCAACAACAACAACATCAATATCACCGGAGTTTCAATGAAAGAGAGGAGGAGA   300
BnaC09G0354200ZS_D1     CCGGCGAGTCAAGAGATTCGTTAACGAGATCAAAGTCGAGCCAGCCTGCAACAACAACAACATCAATATCACCGGAGTTTCAATGAAAGAGAGGAGGAGA   300

BnaC09G0354200ZS_M98    CTCTCCGATGAAACTCAATATCCGGCGACCAGTCGTCACCGTCGTCCACTCAAGGTATCAATCCCCTCCGCTCAAAATGGGAGGAAATTCCGCGGCGTTA   400
BnaC09G0354200ZS_D1     CTCTCCGATGAAACTCAATATCCGACGACCAGTCGTCACCGTCGTCCACTCAAGGTATCAATCCCCTCCGCTCAAAATGGGAGGAAATTCCGCGGCGTTA   400

BnaC09G0354200ZS_M98    GACAACGGCCGTGGGGGAAATGGGCGGCGGAGATTCGAGATCCCGAGCAACGTCGGAGGATTTGGCTTGGTACTTTCGAGACGGCGGAGGAAGCCGCCGT   500
BnaC09G0354200ZS_D1     GACAACGGCCGTGGGGGAAATGGGCGGCGGAGATTCGAGATCCCGAGCAACGTCGGAGGATTTGGCTTGGTACTTTCGAGACGGCGGAGGAAGCCGCCGT   500

BnaC09G0354200ZS_M98    GGTTTATGATAACGCTGCCATTAGACTCCGTGGACCGGACGCTTTAACCAATTTCTCTATACCGCCTCAATCTCAAGAAGAAGAAGAAGAACCGGAACAA   600
BnaC09G0354200ZS_D1     GGTTTATGATAACGCTGCCATTAGACTCCGTGGACCGGACGCTTTAACCAATTTCTCTATACCGCCTCAATCTCAAGAAGAAGAAGAAGAACCGGAACAA   600

BnaC09G0354200ZS_M98    CCGGTTATTGATAAACCGGAAAACAACATTGCAACGACAACAACAACAACAACAACAACATCCAGTTCCGAATCAACCGAAGATTTTCAACATCTTTCAT   700
BnaC09G0354200ZS_D1     CCGGTTATTGATAAACCGGAAAACAACATTGCAACGACAACAACAACAACAACAACAACATCCAGTTCCGAATCAACCGAAGATTTTCAACATCTTTCAT   700

BnaC09G0354200ZS_M98    CTCCTACATCGGTTCTCAATATCCGACCATCAGAAGACATACAACAACCGTTTAAATCAGCTAAACCGGAACCAGAAACTTCAGATGCACCATGGTGGCA   800
BnaC09G0354200ZS_D1     CTCCTACATCGGTTCTCAATATCCGACCATCAGAAGACATACAACAACCGTTTAAATCAGCTAAACCGGAACCAGAAACTTCAGATGCACCATGGTGGCA   800

BnaC09G0354200ZS_M98    TAGCGGGTTTAGTTCCGGTTCAGGTGAATCAGACGATTCGTTCCCTTTGGATACTTCATTTCTCGACAGCTATTTCAACGAATGTCCACCTGAGATTTCA   900
BnaC09G0354200ZS_D1     TAGCGGGTTTAGTTCCGGTTCAGGTGAATCAGACGATTCGTTCCCTTTGGATACTTCATTTCTCGACAGCTATTTCAACGAATGTCCACCTGAGATTTCA   900

BnaC09G0354200ZS_M98    ATATTCGATCAACCAATGGGTCAAGTATTTTTTGAAAATGATGATACATTCAATGATATGTTCTTGGGTGGTGAAATTATGATGATGAACATTGGAGAGG   1000
BnaC09G0354200ZS_D1     ATATTCGATCAACCAATGGGTCAAGTATTTTTTGAAAATGATGATACATTCAATGATATGTTCTTGGGTGGTGAAATTATGATGATGAACATTGGAGAGG   1000

BnaC09G0354200ZS_M98    AGTTTACTTCATCCAGTATCAAAGATATTGGTTCAATGTTCAGTGATTTTGATGATTCATTGATATCAGATTTATTACTGGTTTAA   1086
BnaC09G0354200ZS_D1     AGTTTACTTCATCCAGTATCAAAGATATTGGTTCAATGTTCAGTGATTTTGATGATTCATTGATATCAGATTTATTACTGGTTTAA   1086
```

Supplementary Figure S5 Nucleic acid sequences of *BnaC09G0354200ZS* in rapeseed *M98* and *D1*. Green backgroud, synonymous SNP. Blue backgroud, nonsynonymous SNP.

```
BnaC09G0354200ZS_M98    MMEEYIDFRPLKYTEHKTSVTKYTKKSPAKNPSGETRPDSVRIVRVSVIDPYATDSSSDEEDFLFPRRRVKRFVNEIKVEPACNNNNINITGVSMKERRR   100
BnaC09G0354200ZS_D1     MMEEYIDFRPLKYTEHKTSVTKYTKKSPAKNPSGETRPDSVRIVRVSVTDPYATDSSSDEEDFLFPRRRVKRFVNEIKVEPACNNNNINITGVSMKERRR   100
```

BnaC09G0354200ZS_M98 LSDETQYPATSRHRRPLKVSIPSAQNGRKFRGVRQRPWGKWAAEIRDPEQRRRIWLGTFETAEEAAVVYDNAAIRLRGPDALTNFSIPPQSQEEEEEPEQ 200
BnaC09G0354200ZS_D1 LSDETQYPTTSRHRRPLKVSIPSAQNGRKFRGVRQRPWGKWAAEIRDPEQRRRIWLGTFETAEEAAVVYDNAAIRLRGPDALTNFSIPPQSQEEEEEPEQ 200

BnaC09G0354200ZS_M98 PVIDKPENNIATTTTTTTTTSSSESTEDFQHLSSPTSVLNIRPSEDIQQPFKSAKPEPETSDAPWWHSGFSSGSGESDDSFPLDTSFLDSYFNECPPEIS 300
BnaC09G0354200ZS_D1 PVIDKPENNIATTTTTTTTTSSSESTEDFQHLSSPTSVLNIRPSEDIQQPFKSAKPEPETSDAPWWHSGFSSGSGESDDSFPLDTSFLDSYFNECPPEIS 300

BnaC09G0354200ZS_M98 IFDQPMGQVFFENDDTFNDMFLGGEIMMMNIGEEFTSSSIKDIGSMFSDFDDSLISDLLLV 361
BnaC09G0354200ZS_D1 IFDQPMGQVFFENDDTFNDMFLGGEIMMMNIGEEFTSSSIKDIGSMFSDFDDSLISDLLLV 361

Supplementary Figure S6 Protein sequences of BnaC09G0354200ZS in rapeseed *M98* and *D1*. Blue backgroud, amino acid variant.

BnaC09G03532000ZS_M98 ATGTGGGTTTTGGTTTGTGATCAGGGGTTTATCCTGTTTTGTAGTTATTTTGATGTCTTGATGTCTAAGACACAAATCAACAAAATTGATTTGTTCTTCC 100
BnaC09G03532000ZS_D1 ATGTGGGTTTTGGTTTGTGATCAGGGGTTTATCCTGTTTTGTAGTTATTTTGATGTCTTGATGTCTAAGACACAAATCAACAAAATTGATTTGTTCTTCC 100

BnaC09G03532000ZS_M98 TCTACAGATTTCGTCAGACGGTTCCTTCACACTCGGTATCATCAATTTCATCAAAAACTAACGTTCCTTTACACTCGGTTGGAGCAGAGATCGTTAAAAG 200
BnaC09G03532000ZS_D1 TCTACAGATTTCGTCAGACGGTTCCTTCACACTCGGTATCATCAATTTCATCAAAAACTAACGTTCCTTTACACTCGGTTGGAGCAGAGATCGTTAAAAG 200

BnaC09G03532000ZS_M98 AGCACTGAGTTACCCACTGAAATTGACAGCAAAGAATGCTGGTGTCAACGGAAGCGTTGTCAGCGAGAAGGTTGTGAGATGTTGCTTGGGACATGCGGAC 300
BnaC09G03532000ZS_D1 AGCACTGAGTTACCCACTGAAATTGACAGCAAAGAATGCTGGTGTCAACGGAAGCGTTGTCAGCGAGAAGGTTGTGAGATGTTGCTTGGGACATGCGGCC 300

BnaC09G03532000ZS_M98 TCGGTTGCAAAAACGTTCTTTATGTCTAACTGTGTGGTTGTTGAGAAAGGAGCCAACACCAGTTCCTACTGGCAACCCAATGAACAATTCAGGATATGGA 400
BnaC09G03532000ZS_D1 TCTGTTGCAAAAACGTTCTTTATGTCTAACTGTGTGGTTGTTGAGAAAGGAGCCAACACCAGTTCCTACTGGCAACCCAATGAACAATTCAGGATATGGA 400

BnaC09G03532000ZS_M98 TACTGAGAAAGACCAGCCGGATTTACTCTTGA 432
BnaC09G03532000ZS_D1 TACTGAGAAAGACCAGCCGGATTTACTCTTGA 432

Supplementary Figure S7 Nucleic acid sequences of *BnaC09G0353200ZS* in rapeseed *M98* and *D1*. Green backgroud, synonymous SNP. Blue backgroud, nonsynonymous SNP.

BnaC09G03532000ZS_M98 MWVLVCDQGFILFCSYFDVLMSKTQINKIDLFFLYRFRQTVPSHSVSSISSKTNVPLHSVGAEIVKRALSYPLKLTAKNAGVNGSVVSEKVVRCCLGHAD 100
BnaC09G03532000ZS_D1 MWVLVCDQGFILFCSYFDVLMSKTQINKIDLFFLYRFRQTVPSHSVSSISSKTNVPLHSVGAEIVKRALSYPLKLTAKNAGVNGSVVSEKVVRCCLGHAA 100

BnaC09G03532000ZS_M98 SVAKTFFMSNCVVVEKGANTSSYWQPNEQFRIWILRKTSRIYS 143
BnaC09G03532000ZS_D1 SVAKTFFMSNCVVVEKGANTSSYWQPNEQFRIWILRKTSRIYS 143

Supplementary Figure S8 Protein sequences of BnaC09G0353200ZS in rapeseed *M98* and *D1*. Blue backgroud, amino acid variant.

BnaC09G0356600ZS_M98 ATGATCCAAAACGAATTTGAGATAGATAACTCAACGGTTGTCCGTTGTCCGATGGCAGAAGAAAAACTCAAAACACTCATATCATTCATGGCTTCGCTTC 100
BnaC09G0356600ZS_D1 ATGATCCAAAACGAATTTGAGATAGATAACTCAACGGTTGTCCGTTGTCCGATGGCAGAAGAAAAACTCAAAACACTCATATCATTCATGGCTTCGCTTC 100

BnaC09G0356600ZS_M98 CTGTCCAGTTCTCCAGGAATCATCATCTCTCTACCCCCAATTTCTCCAGTAGATTCCGCCGTTCCACGGAGCAGAGATCTTTTGTAGCTCTTGTTCATTG 200
BnaC09G0356600ZS_D1 CTGTCCAGTTCTCCAGGAATCATCATCTCTCTACCCCCAATTTCTCCAGTAGATTCCGCCGTTCCACGGAGCAGAGATCTTTTGTAGCTCTTGTTCATTG 200

BnaC09G0356600ZS_M98 CTCCGCTAGAGAAAACGGAGACGCTGATGTTCGAGGAATCAAGAAGAGTTTGTTTCCTGTTAAGGAGCTTGGATGTCTCGCTTGCGCCGCTCTTTCCGCT 300
BnaC09G0356600ZS_D1 CTCCGCTAGAGAAAACGGAGACGATGATGTTCGAGGAATCAAGAAGAGTTTGTTTCCTGTTAAGGAGCTTGGATGTCTCGCTTGCGCCGCTCTTTCCGCT 300

BnaC09G0356600ZS_M98 TTCACTCTTACATTAGCTTCTCCCGTCATTGCTGCTAACCAGAGACTTCCTCCGCTTTCAACAGATCCAACACGGTGTGAACAAGCTTTTGTTGGTAACA 400
BnaC09G0356600ZS_D1 TTCACTCTTACATTAGCTTCTCCCGTCATTGCTGCTAACCAGAGACTTCCTCCGCTTTCAACAGATCCAACACGGTGTGAACAAGCTTTTGTTGGTAACA 400

BnaC09G0356600ZS_M98 CGATAGGTCAAGCAAACGGGGTCTATGACAAGCCACTCGATCTCAGGTTCTGCGACTACACAAACGATCAGTCCAATCTCAAAGGCAAGACTCTCTCTGC 500
BnaC09G0356600ZS_D1 CGATAGGTCAAGCAAACGGGGTCTATGACAAGCCACTCGATCTCAGGTTCTGCGACTACACAAACGATCAGTCCAATCTCAAAGGCAAGACTCTCTCTGC 500

BnaC09G0356600ZS_M98 AGCCTTGATGTCAGGCGCCAAGTTTGATGGTGCTGACATGACTGAAGTCGTTATGTCAAAAGCTTACGCCGTTGGAGCAAGCTTCAAGGGGGTGAATTTC 600
BnaC09G0356600ZS_D1 AGCCTTGATGTCAGGCGCCAAGTTTGATGGTGCTGACATGACTGAAGTCGTTATGTCAAAAGCTTACGCCGTTGGAGCAAGCTTCAAGGGGGTGAATTTC 600

BnaC09G0356600ZS_M98 TCGAATGCTGTCATCGACCGGGTGAACTTCGGGAAGTCGGATCTAAAAGGAGCTGTGTTCAAGAACACGGTGTTGTCAGGTTCGACATTCGACGAGGCAA 700
BnaC09G0356600ZS_D1 TCGAATGCTGTCATCGACCGGGTGAACTTCGGGAAGTCGGATCTAAAAGGAGCTGTGTTCAAGAACACGGTGTTGTCAGGTTCGACATTCGACGAGGCAA 700

BnaC09G0356600ZS_M98 ACCTGGAGGATGTGGTCTTTGAGGACACCATCATTGGTTACATAGACCTTCAGAAGATTTGTAGGAACGAGACTATTAACGAAGAAGGAAGACTCGTCTT 800
BnaC09G0356600ZS_D1 ACCTGGAGGATGTGGTCTTTGAGGACACCATCATTGGTTACATAGACCTTCAGAAGATTTGTAGGAACGAGACTATTAACGAAGAAGGAAGACTCGTCTT 800

BnaC09G0356600ZS_M98 GGGTTGCAGATAA 813
BnaC09G0356600ZS_D1 GGGTTGCAGATAA 813

Supplementary Figure S9 Nucleic acid sequences of *BnaC09G0356600ZS* in rapeseed *M98* and *D1*. Blue backgroud, nonsynonymous SNP.

```
BnaC09G0356600ZS_M98    MIQNEFEIDNSTVVRCPMAEEKLKTLISFMASLPVQFSRNHHLSTPNFSSRFRRSTEQRSFVALVHCSARENGDADVRGIKKSLFPVKELGCLACAALSA  100
BnaC09G0356600ZS_D1     MIQNEFEIDNSTVVRCPMAEEKLKTLISFMASLPVQFSRNHHLSTPNFSSRFRRSTEQRSFVALVHCSARENGDDDVRGIKKSLFPVKELGCLACAALSA  100

BnaC09G0356600ZS_M98    FTLTLASPVIAANQRLPPLSTDPTRCEQAFVGNTIGGQANGVYDKPLDLRFCDYTNDQSNLKGKTLSAALMSGAKFDGADMTEVVMSKAYAVGASFKGVNF  200
BnaC09G0356600ZS_D1     FTLTLASPVIAANQRLPPLSTDPTRCEQAFVGNTIGGQANGVYDKPLDLRFCDYTNDQSNLKGKTLSAALMSGAKFDGADMTEVVMSKAYAVGASFKGVNF  200

BnaC09G0356600ZS_M98    SNAVIDRVNFGKSDLKGAVFKNTVLSGSTFDEANLEDVVFEDTIIGYIDLQKICRNETINEEGRLVLGCR  270
BnaC09G0356600ZS_D1     SNAVIDRVNFGKSDLKGAVFKNTVLSGSTFDEANLEDVVFEDTIIGYIDLQKICRNETINEEGRLVLGCR  270
```

Supplementary Figure S10 Protein sequences of BnaC09G0356600ZS in rapeseed *M98* and *D1*. Blue backgroud, amino acid variant.

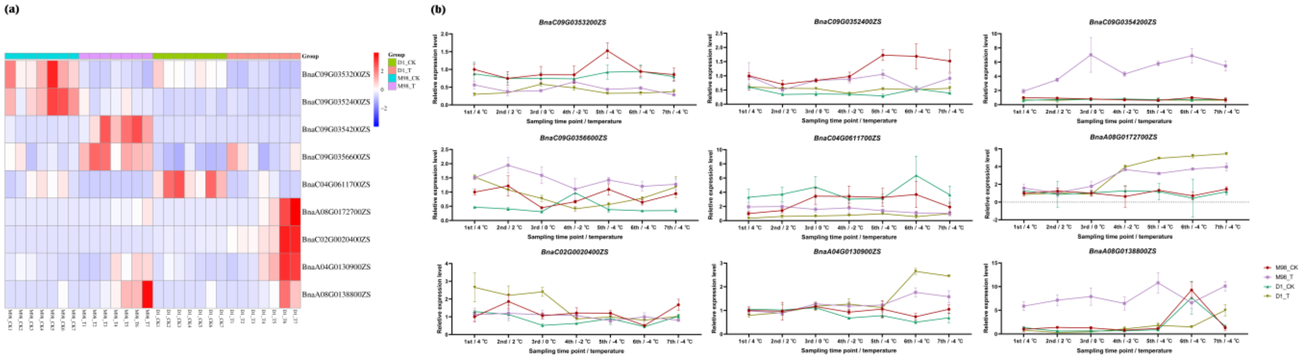

Supplementary Figure S11 Expression patterns of candidate genes. (a)RNA-seq expression heatmap of candidate genes. The legend indicates that the expression level FPKM is normalized by row. (b) qRT-PCR analysis of candidate genes during low temperature treatment. Expression level of each gene in M98-CK1 was set to 1 (b) Expression level of *BnACTIN* was used as control reference. Each point of the line chart shows the average value of three repeats, and each error bar represents one standard error. CK: control; T, low-temperature treatment.

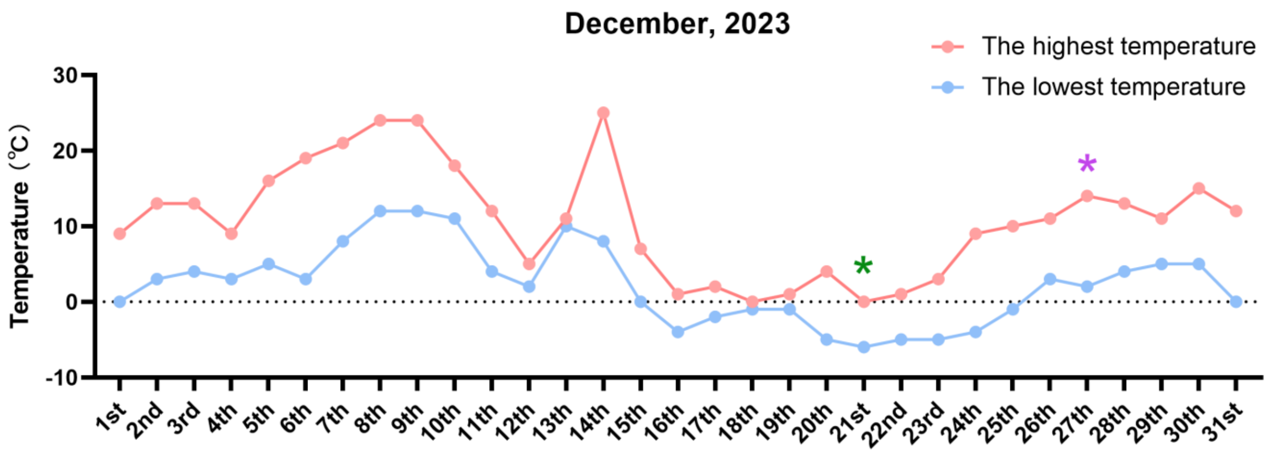

Figure S12 Historical temperature during the coldest month in Deqing Rapeseed Farm, spanning from 2023 to 2024. The green asterisk represents the coldest date, and the purple asterisk represents the date of phenotypic identification.

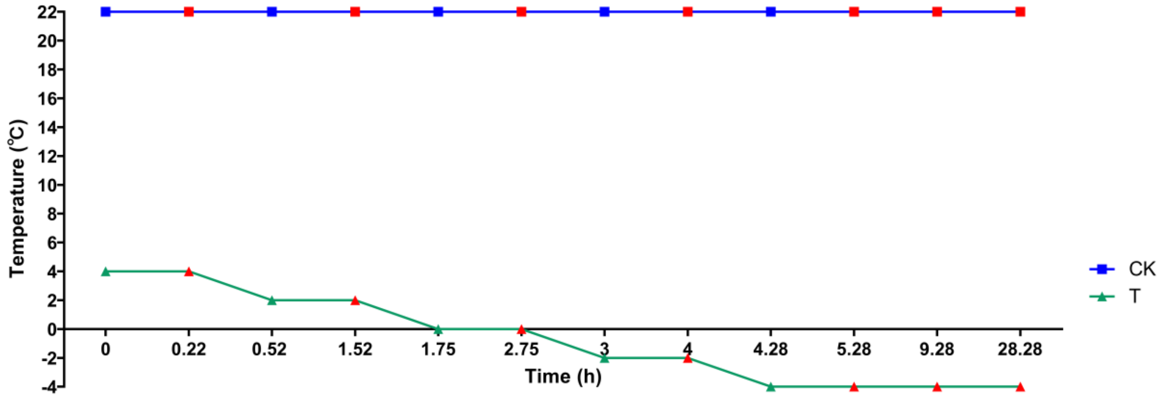

Supplementary Figure S13 Sampling time point. The red boxes and triangles in the figure represent the time points sampled, named 1st-7th sampling time point from left to right. CK: control; T, low-temperature treatment.
